# In the margins of stigma: health inequalities among Bulgarian Roma in a post-COVID-19 UK

**DOI:** 10.1136/bmjgh-2024-015686

**Published:** 2024-11-04

**Authors:** Iliana Sarafian, Alice Robinson, Assen Christov, Aleksandra Tarchini

**Affiliations:** 1 Firoz Lalji Institute for Africa, The London School of Economics and Political Science, London, UK; 2 The London School of Economics and Political Science, London, UK

**Keywords:** COVID-19, health policies and all other topics

## Abstract

The COVID-19 pandemic had a disproportionate impact on minoritised ethnic groups in the UK, including newly arrived Roma communities. Employing ethnographic and participatory methods, this study illustrates how systemic barriers, including precarious employment and overcrowded housing, coupled with strategies of identity concealment to avoid stigma, severely restrict access to healthcare among Bulgarian Roma communities in the UK. Drawing from fieldwork in Leicester and London, the research reveals how the pandemic amplified the vulnerabilities of Roma populations, directly linking the effects of the pandemic with broader sociopolitical dynamics, including the uncertainties and discrimination associated with Brexit. The findings point to the critical role of community, mutual and familial support networks as essential survival strategies. However, these social networks are also increasingly depleted, revealing the fragility and limits of informal communal resources. The study calls for the development of inclusive health strategies sensitive to the socio-economic and political complexities affecting marginalised communities in the UK and beyond.

**Summary box**

- Roma populations in the UK face significant barriers to healthcare access due to structural discrimination, language issues and precarious living conditions, yet they are often labelled as ‘hard to reach’ and remain under-represented in research.
- Ethnographic research reveals how Roma communities adopt strategies of invisibility to navigate stigma, which further exacerbates their healthcare access challenges.
- The study advocates for more inclusive health policies that account for Roma experiences and address structural barriers.
- Recognising the importance and fragility of mutual support networks within Roma communities could inform more culturally sensitive and effective health interventions.

## Introduction

 Throughout the COVID-19 pandemic, minoritised ethnic groups in the UK, including Roma populations, faced heightened risks of morbidity and mortality,^1^,^4^ exacerbating pre-existing health inequalities. These social processes have spurred scientific investigations into potential causes, including genetic and epidemiological factors. However, conventional analyses of ethnic health disparities often amalgamate ethnicities, obscuring the intricate nature of health disparities. Moreover, the postpandemic recovery narrative has predominantly centred on economic metrics such as growth and productivity, neglecting the myriad ethnic, racial and socioeconomic challenges that persist in a post-COVID-19 world.

This study explores the health experiences of Roma populations in the UK, who are often labelled as ‘hard to reach’ in policy and health interventions but are seldom engaged in research.^5^ The pandemic intensified racist anti-Roma attitudes across Europe, blaming Roma for spreading the virus and labelling them as ‘hesitant’, ‘resisting’ or ‘non-compliant’ with public health measures, including vaccination campaigns,^6^,^12^ and led several states to implement securitised and militarised measures targeting Roma communities.^6 8 10^ By adopting a qualitative lens, our research illustrates how social and political inequalities and continued everyday stigmatisation directly influence health outcomes.

In the UK, despite clear evidence of healthcare access disparities, research on the health experiences of Roma populations remains limited.^13^ This was exemplified in the Evidence for Equality National Survey (EVENS) in 2023, a quantitative study on the deep-rooted structural and institutional racism perpetuating health disparities among ethnic minorities, including Roma communities in the UK.^14^ The ethnographic and participatory data collected by our research team complements the EVENS study and the ‘Routes’ report commissioned by the Department of Health and Social Care,^12^ further suggesting that Roma have greater difficulties compared with other groups in accessing and using healthcare services.

We align our empirical results with the essential principles of critical medical anthropology, which centre on the structural determinants of health: the social, political and economic inter-relationships shaping health outcomes and experiences of illness.^15 16^ Our study underscores the importance of moving beyond a siloed focus on biomedicine, individual behaviours, risk factors or economic circumstance. Instead, we emphasise the need to understand the ‘local biologies’ in which our informants live^17^ and how power dynamics contribute to varying health outcomes among marginalised groups.^18 19^

The methodological objective of the study is to present first-hand accounts of Roma encounters with the pandemic and their subsequent life adjustments, thereby underlining the manifold socioeconomic and health repercussions experienced by our participants. These findings contribute to a broader understanding of socioeconomic stratification of health outcomes and provide valuable insights for guiding postpandemic recovery and preparedness efforts. A comprehensive approach building on available national statistics and employing ethnographic methodologies can inform policymaking effectively, offering an understanding of the intersecting dimensions of health inequalities faced by minoritised communities.

### Study population, settings, methods and data collection

This analysis piece is based on ethnographic fieldwork conducted as part of a research project titled ‘Stigma in a post-COVID-19 world: ethnographies of health among Roma communities in the UK’. The project built on an earlier study on vaccine hesitancy among minoritised groups, including Roma communities in Italy,^10^ and on the first author’s long-standing ethnographic fieldwork with Roma communities in Bulgaria.^20^ We set out to explore communal views of and responses to the COVID-19 crisis among Roma communities and to communicate how stigma is experienced and contested within Roma groups and in their relationship with the healthcare system in the UK.

In the UK, the umbrella term ‘Gypsy, Roma and Travellers’ encompasses diverse subgroups, with varied names, histories and languages, as well as differences in religion, geographic location and preference for self-identification.^21^ In this paper, following Brown *et al*,^22^ we use the term ‘Roma’ to refer to people of Roma origin who have migrated from Central and Eastern Europe to the UK since the 1990s, while acknowledging the multiplicity of identities and experiences subsumed within this label. Our research focused on the experiences of Roma individuals in two cities (London and Leicester), who have migrated to the UK from Bulgaria.

Estimating the population size of migrant Roma in the UK poses challenges due to long-standing exclusion and discrimination, underscoring the need for cautious interpretation of available statistics.^22^ Despite the first inclusion of a specific Roma category in the 2021 census, the count remains a contentious issue, revealing the complexities of accurately capturing Roma populations’ size and composition. Ethnographic methodologies, including participant observation and mapping, offered insights into how health inequalities interact with sociocultural, economic and political contexts.

The data collection team comprised four researchers, including one anthropologist, two Roma citizen scientists and a postdoctoral researcher in migration and international development. The two citizen scientists were recruited through the existing personal and professional networks of the lead author and were an integral part of the social processes studied. The two Roma citizen scientists played a crucial role by improving access, building trust with respondents, co-designing the study, conducting interviews, leading workshops and co-authoring publications. Three of the four researchers spoke Bulgarian and Romani, which helped overcome language and cultural barriers, enabling strong rapport with participants. The research team also remained critically aware of potential biases, carefully balancing insider perspectives with reflexive analysis to ensure the integrity of the findings.

The research team employed a community-based participatory research approach previously developed by the COVID and Care Research Group, led by Professor Laura Bear at the London School of Economics.^23^ This approach was chosen to ensure a community-embedded understanding, particularly given the stigma and discrimination faced by Roma communities. Data collection was based on semi-structured interviews, with participants being asked about their primary health concerns. Two follow-up workshops were organised in Leicester and London, where research themes were identified through dialogic inquiry with research participants. This strategy ensured culturally sensitive engagement and direct communal input into the analysis of the data. To manage the data, we used Excel to maintain codebooks, allowing for a systematic and transparent coding process. Although we did not follow a strict grounded theory approach, the coding was iterative and informed by insights from the workshops, ensuring a reflexive and inclusive analysis.

The research took place between September 2022 and September 2023. London and Leicester were selected to capture diverse socioeconomic urban settings and their impact on health inequalities. Leicester is one of the fastest-growing cities in the UK with relatively high levels of poverty and deprivation and life expectancy lower than in England overall.^24^ Similarly, interviews in London were conducted within the Borough of Haringey with high deprivation levels and significant gaps in life expectancy.^25^ In Leicester, a total of 52 participants took part in interviews and focus groups, with ages ranging from 23 to 65 years. In London, interviews were conducted with 40 individuals aged between 19 and 60 years. The duration of participants’ residence in the UK varied from 1 month to 20 years.

### Long-standing health disparities and stigma

A significant body of evidence suggests that Roma groups across Europe have poorer health and higher mortality than non-Roma, including higher rates of long-term illness, and face numerous barriers in accessing adequate healthcare,^26^,^29^ particularly when compared with other ethnic groups. Roma, Gypsy and Traveller communities in the UK have a life expectancy that is 10–12 years shorter than the national average,^30 31^ while across Europe, the life expectancy gap between Roma and non-Roma can reach 20 years.^32^

In 2023, a study revealed pervasive racial discrimination experienced by Roma communities in the UK before and during the COVID-19 pandemic, including by the police, in education, employment and public spaces.^33 34^ Moreover, chronic illness among Roma was found to be highly prevalent. Roma men were five times more likely to suffer from two or more physical conditions (such as high blood pressure, diabetes, heart disease, lung disease and cancer) compared with white British men.^4 35^ These conditions are often exacerbated by delays in accessing acute care, partly due to systemic barriers such as lack of healthcare registration, language difficulties and mistrust of medical services.^11 12^ As a result, Roma populations in the UK frequently experience worse outcomes, including advanced disease progression by the time treatment is sought, compared with their non-Roma counterparts.^26^ Adding to these structural inequalities, the COVID-19 pandemic coincided for Roma communities in the UK with the uncertainties and discrimination towards migrants resulting from the UK’s exit from the EU^36 37^ and hostile environment policies that have restricted migrants’ access to healthcare, intensifying fear and mistrust.^12^ In line with this, our research participants overwhelmingly made attempts to hide aspects of their identities from employers, colleagues, neighbours and health professionals to avoid discrimination associated with being identified as Roma. In the words of one woman from Leicester working in a sewing factory: “*My employers don’t know that I’m a Roma, I think they would get rid of me if they knew it*”*.* Research participants believed that being labelled as Roma results in being perceived as deceitful or inferior.

It is well established that stigma is a major driver of health inequalities, undermining access to health services, housing and employment and generating physiological stress.^37^ A history of experiencing stigma can directly impact an individual’s health, creating physiological effects and preventing people from accessing healthcare. To navigate stigma, our interlocutors often adopted strategies of invisibility, avoiding identifying themselves as Roma, instead allowing others to classify them based on characteristics such as nationality, typically Bulgarian or Eastern European. One respondent in Leicester shared: “*I do not say that I am a gypsy, so that they do not change their opinion and trust in me*”. Similar patterns of living ‘veiled in invisibility’ were observed among Slovakian Roma migrants residing in London.^38^ Among our participants, the reluctance to disclose their Roma identity stemmed from past encounters with discrimination and social exclusion in Bulgaria, coupled with an awareness of the discrimination faced by Roma, Gypsy and Traveller communities in the UK and the general antimigrant sentiments invoked after Brexit. Participants recounted instances where neighbours and employers exhibited prejudice on discovering their Roma identity. In the words of another respondent: “*I don’t want people to know that I am Roma. If you say the word ‘Gypsy’ people think that you are a scam, rubbish, the lowest of the low in society*”.

On the other hand, our respondents also grappled with feelings of shame and guilt for concealing their identity. This internal conflict reflected the internalisation of societal prejudices and self-stigmatisation, including avoiding contact with health providers and delaying seeking healthcare. Self-stigma combined with distrust in public services due to historical and ongoing discrimination resulted in limited engagement with public health initiatives and a lack of awareness about available healthcare services and entitlements.^39 40^ Most of our interviewees believed that health practitioners would not pay attention to their health needs due to language barriers.

Existing research has found that migrants in England were less likely than non-migrants to access primary care before the pandemic and that this gap grew during the first year of the pandemic,^41^ with digitalisation and virtual consultations exacerbating inequalities in access to healthcare.^42^ Our research participants overwhelmingly shared that they were not registered with a general practitioner (GP), or that if they were registered, they rarely or never sought an appointment. Instead, if unwell, they sought treatment at home, with support from relatives. One man in Leicester told us: “*We have a GP in the UK, but we do not go to the doctor, it takes too long to arrange an interpreter; we treat ourselves with medicines from Bulgaria. We also share medicines with others who need them*”. Most informants also reported travelling to Bulgaria to seek medical care. Yet, on returning to Bulgaria, they encountered significant private healthcare expenses due to lost health insurance rights. Regaining these rights necessitated a substantial payment to cover missed contributions, a requirement that was unattainable for many.^43^ Illustrating the severity of this issue, a Roma woman employed at a sewing company in Leicester disclosed that she had exhausted her savings and resorted to borrowing money from loan sharks to finance her cancer treatment in Bulgaria before returning to low-paid informal employment in the UK. Additionally, challenges such as precarious work, long working hours, lack of stable housing and non-registration with a GP, further hindered access to healthcare.^41 44^

### Precarious employment

In general, the experience of Roma migrants in the labour market in the UK is under-researched.^45^ Most of our research participants worked in short-term employment, on zero-hours contracts or in the gig economy without union protection, and, like many others in a similar situation, had greater difficulties in reducing the risk of exposure to or self-isolating from the COVID-19 virus.^1^ Government measures aimed at aiding businesses and employees throughout the COVID-19 crisis were misaligned with the actual conditions of casual employment, gig economy labour, suppressed wages and the existing challenges of poverty and deprivation among working individuals in the UK.^46^

Both in Leicester and London, our informants were predominantly engaged in precarious work, characterised by low pay, long hours and difficult working conditions, often involving heavy or intensive manual labour, including warehouse, factory and cleaning work. They were susceptible to labour exploitation, a situation also found in previous research.^12 47^ The formality of employment ranged from informal cash payments to more structured work with insurance coverage. Some of our informants worked without contracts for well below the UK minimum wage. Regional disparities were evident, with London offering relatively higher pay and more diverse job opportunities compared with Leicester, however, accommodation costs in London were higher. With no financial support and no savings to fall back on, some felt that they had no choice other than to continue working during lockdowns. As one interviewee in London noted, they had ‘*to work to be able to live’* and often ‘*behind locked doors’*. Those who fell sick stayed at home and were not paid. These findings resonate with recent research illustrating how spatial aspects of precarious employment conditions during the pandemic increased health risks, both at work and at home, especially in overcrowded living situations.^47^

Our findings support existing research on the challenges faced particularly by precariously employed and low-income migrants during the COVID-19 pandemic.^12^ These conditions were exacerbated by exploitative practices perpetrated by employers, including underpayment and wage withholding. One woman shared the following: “*I am a seamstress. My job is standardised on documents, but it is not really. Often, we get items returned from the previous day for no reason. We can work for hours, and we don’t get paid*”. Our findings reveal that a significant barrier to seeking redress for exploitation was the limited English proficiency among most of our interviewees, accentuating that language and legal obstacles contribute to the perpetuation of work exploitation. Additionally, the dependence on employers for housing further entrenched individuals in exploitative employment conditions. Research has noted that such interconnectedness of employment and housing severely constrained workers’ ability to mitigate health risks, creating a cycle where precarious work and living conditions mutually reinforce vulnerability to exploitation and disease.^48^ In several instances, informants disclosed that their employer was also their housing guarantor, or had connections with the guarantor, which meant that attempting to exit exploitative work situations could simultaneously result in losing their accommodation. This link between employment and accommodation significantly restricted their options for leaving adverse work conditions. One woman from Leicester explained, “*My boss is my guarantor, which prevents me from leaving the company even though I don’t like my job*”. This situation was further complicated in environments of overcrowded housing, where the ability to self-isolate or reduce exposure to COVID-19 was severely limited.

### Overcrowded housing and precarious familial support

During the pandemic, research uncovered a significant disparity in living conditions, with 60% of Roma in the UK experiencing overcrowded housing.^4^ Our research participants consistently described facing overcrowded living conditions, often sharing limited space with multiple individuals. Families shared small apartments or houses, with numerous occupants struggling for access to limited facilities. In many cases, there were between 8 and 14 people sharing a three-bedroom or four-bedroom flat. The impact of overcrowded housing on the well-being of our research respondents was evident, and many shared that their accommodation conditions had caused stress, exhaustion and adverse health effects, including exacerbated asthma.

All participants were living in privately rented accommodation, and the quality of housing was uniformly reported as substandard, with prevalent issues such as dampness, mould and neglect by landlords despite regular rent payments. One woman in London shared: “*I am constantly stressed. I want to eat healthy, but I often don’t. I work and live in bad conditions; I don’t have a choice*”. The inadequate living conditions often led to families being separated, with children sent to live with relatives in Bulgaria while parents remained in the UK to work and send financial remittances to them. There were regional disparities in the housing conditions of our interlocutors, with research respondents in Leicester experiencing higher levels of overcrowding compared with London, although accommodation challenges persisted in both locations. Some participants also faced discrimination in the UK, as one respondent shared: “*When our neighbours understood that we are Roma, one of them came to warn us that they don’t want any problems*”.

Throughout the pandemic, our informants relied heavily on family and community networks to overcome challenges related to health, employment and accommodation. The support network extended beyond immediate relatives to include extended family, community members and the local informal Roma churches, which were the main ‘social infrastructure’^46^ of support for the communities of our research. One man in London told us: “*When Roma people died of COVID, our churches were collecting money to help families who often did not have the money to transport the bodies of the deceased to be buried back home or to be cremated*”. This collective support system was vital in providing financial assistance to one another, healthcare through the exchange of medicines for those struggling with ill health and help with daily tasks. These existing and new forms of mutuality were also integral for the general well-being of our informants, who often cited ‘*Sunday morning or church time*’ as their only opportunity to take time off work and reconnect with family and friends. These kinship ties were essential for their survival and played a pivotal role in mitigating the difficulties associated with migration and the distress of family separation, emphasising the indispensable role of communal formations in tackling health inequalities.

## Conclusion

The compounded impact of occupational instability and overcrowded, substandard housing conditions exacerbates health vulnerabilities and underscores a broader theme of structural violence and inequity deeply embedded within societal systems.^19^ The findings align with recent studies on Roma communities and precariously employed migrants during COVID-19,^12 46^ as well as quantitative data on Roma health in the UK.^14^ Central to the research findings is the way stigma and marginalisation are experienced by Roma participants through identity concealment, further entrenching cycles of exclusion and disadvantage. The narratives of Bulgarian Roma in the UK reveal the profound challenges of navigating crisis amid entrenched structural inequalities. These experiences, framed against the backdrop of heightened pandemic-related health risks, are emblematic of the broader, systemic challenges that perpetuate health disparities among marginalised communities in the UK.

Our research advocates for the critical need for holistic, inclusive public health strategies that address the root causes of health disparities by recognising the interconnectedness of socio-economic conditions, systemic discrimination and health outcomes. Health agencies, the National Health Service and government bodies must prioritise targeted interventions that consider the complex sociopolitical dynamics affecting Roma communities. Implementing community-driven approaches are essential to ensuring that health messages resonate and are accessible to communities experiencing stigma. Crucially, as researchers, we conclude that ethnographic data and varied forms of community knowledge should be leveraged for outreach, particularly integrating formal and informal social infrastructures that are vital to these communities.

## Data Availability

All data relevant to the study are included in the article.
